# Prognostic and predictive value of radiomics-based imaging features in patients with colorectal liver metastasis receiving radioembolisation in first-line setting

**DOI:** 10.1016/j.ejro.2026.100750

**Published:** 2026-04-15

**Authors:** Osman Öcal, Anna Theresa Stüber, Gizem Abacı, Moritz Wildgruber, Nabeel Mansour, Sinan Deniz, Daniel Puhr-Westerheide, Matthias Philipp Fabritius, Jens Ricke, Michael Ingrisch, Max Seidensticker

**Affiliations:** a Department of Diagnostic and Interventional Radiology, Heidelberg University Hospital, Heidelberg, Germany; b Department of Radiology, University Hospital, LMU Munich, Munich, Germany; c Department of Statistics, LMU Munich, Munich, Germany; d Munich Center for Machine Learning (MCML), Munich, Germany

**Keywords:** Liver, Colorectal, Radioembolisation, Radiomics, Predictive

## Abstract

**Purpose:**

To evaluate the prognostic and predictive value of radiomics-based imaging markers in colorectal liver metastasis treated with chemotherapy alone or combined with selective internal radiation therapy in the first-line setting.

**Methods:**

This was a post-hoc retrospective analysis of the randomized controlled SIRFLOX trial. 491 patients (333 male, median age 63 [range, 28–83] years) with available baseline Computed Tomography (CT) images were included in this analysis. All lesions were segmented automatically in baseline CT with an nnU-net and evaluated against manual segmentation of 80 patients. Quantitative features of tumor segmentations were computed using PyRadiomics. Least Absolute Shrinkage and Selection Operator (LASSO) regression was used to identify relevant prognostic factors, and potential predictive factors were modeled as interaction with the treatment arm.

**Results:**

239 patients had been randomized to FOLFOX alone arm, and 252 patients to the experimental arm. There was no difference in overall survival between treatment arms. A Cox proportional hazards model with LASSO regularization identified 20 prognostic factors. In addition to seven clinical parameters and eight radiomics-based prognostic markers, the LASSO model identified five interaction effects with treatment, highlighting two radiomics features, "shape - Maximum2DiameterSlice" and "glrlm - RunEntropy," as particularly relevant. When patients were categorized into two risk groups based on the model’s survival predictions ≥ 50%, patients with high-risk had significantly shorter overall survival than the low-risk group (p < 0.001).

**Conclusion:**

Radiomics-based imaging features of liver metastases in pretreatment CT images can identify colorectal cancer patients with poor outcome and potential benefit from combined therapies.

## 1 Introduction

Colorectal cancer was the second leading cause of cancer-related mortality in 2020, and liver metastases are the most common reason for death in these patients [1]. Most patients with liver metastases are not eligible for resection, and systemic therapy (FOLFOX or FOLFIRI) is the first-line treatment of unresectable metastases [2]. In addition to systemic therapies, a number of liver-directed locoregional therapies have been developed to improve the outcome of patients with unresectable liver metastases. Selective internal radiation therapy (SIRT) is a transcatheter therapy delivering radionuclide embedded microspheres to the liver tumors with a high dose ratio of tumor to liver parenchyma with promising results in the initial studies [3], [4], [5]. However, the addition of SIRT to recent systemic therapies did not meet the primary objective of progression-free survival in the SIRFLOX trial [6]. Furthermore, a combined analysis of three phase 3 randomized controlled trials evaluated the additional value of SIRT to FOLFOX regimen showed no survival benefit of additional SIRT [7], except for the patients with right-sided primary tumor [8]. Thus, SIRT in combination with chemotherapy is not recommended in the first line in patients with colorectal cancer,despite improved hepatic progression-free survival (hPFS) in SIRFLOX trial (20.5 vs. 12.6 months). Recently, the EPOCH trial showed a significantly longer PFS and hPFS in patients receiving SIRT combined with the second line chemotherapy compared to chemotherapy alone [9]. This situation underlines the need for additional markers for patient selection to determine the exact role of SIRT in colorectal cancer.

Imaging plays a crucial role in diagnosis and treatment decisions in colorectal cancer and radiologic images contain much more information related to tumor pathophysiology and genomic profile. This information can be exposed through quantitative image analyses (radiomics) and guide treatment selection. For example, it has been shown that the radiomics signature of the primary lesion was correlated with mutation profile in patients with colorectal cancer, while all clinical parameters failed to predict these mutations [10]. Similarly, the prognostic value of texture analysis of colorectal cancer metastasis has been shown in different clinical settings. [11], [12].

This post-hoc analysis of the SIRFLOX trial aimed to evaluate the potential prognostic and predictive value of radiomics-based imaging parameters to identify subgroups benefiting from additional SIRT in patients with metastatic colorectal cancer.

## 2 Materials and methods

### 2.1 Study population

The present study was performed within the cohort of SIRFLOX trial (NCT00724503), a randomized-controlled multicenter phase-III trial evaluated the addition of SIRT to systemic chemotherapy with modified FOLFOX (mFOLFOX6) [6]. The main inclusion criteria of the trial were age> 18, histologically confirmed adenocarcinoma of the colon or rectum with chemotherapy-naive non-resectable liver-only or liver-dominant metastases, WHO performance status of 0–1, and a life expectancy of more than three months. For inclusion in this study, the presence of pretreatment portovenous phase Computed Tomography (CT) images was required. Thus, out of 530 patients randomized in the SIRFLOX trial, 32 who did not receive the allocated treatment and 7 with missing portal venous phase CT images were excluded; 491 patients with available baseline portal-venous phase CT images were included in this analysis.

CT image acquisition guidelines of SIRFLOX trial have been summarized in supplementary table 1. All patients underwent contrast-enhanced CT using 80–160 mL non-ionic contrast agent injected with power injector. The slice thickness of 2 mm was preferred, although up to 5 mm was accepted.

Patients were randomized in a 1:1 ratio to treatment with a mFOLFOX6 regimen alone or mFOLFOX6 plus SIRT with 90 Y-labelled resin microspheres. Chemotherapy was continued until disease progression. In addition, patients could receive bevacizumab at the investigator's discretion from cycle 1 in the control arm and from cycle 4 onwards in the SIRT arm, which is also used in patient stratification.

Patients randomized to the SIRT arm underwent baseline hepatic angiography, and a liver-to-lung shunt was calculated with nuclear medicine scan to assess suitability to receive SIRT. The patient's body surface area, percentage of liver tumor involvement, and magnitude of liver-to-lung shunting were used to determine the administered activity. SIRT was given in a single procedure on day 3 – 4 of cycle 1 or cycle 2 of mFOLFOX6 treatment. Patients were assessed by CT every 8 – 12 weeks until hepatic progression using Response Evaluation Criteria in Solid Tumors (RECIST) 1.1. by two separate and independent reading sessions by radiologists (at least 15 years of experience) blinded to the study arm. Other follow-up assessments included clinical assessment at every CT follow-up. All patients were followed up until death, or to the end of the study.

### 2.2 Image preprocessing

**Fig. 1:**
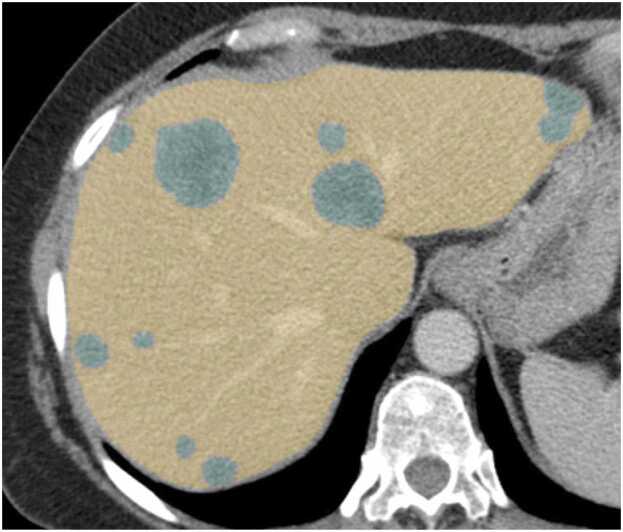
An example case for segmentation. All metastatic lesions (green) in the liver (yellow) have been segmented for the analysis

CT images were rescaled to 1 × 1 × 1 mm³ isotropic spatial resolution. Intrahepatic metastases were segmented using the pre-trained nnU-net, with manual segmentation of 80 randomly selected datasets by a radiologist of seven years of experience (Fig. 1). nnU-net segmentations were assessed against this reference standard using Dice scores with a mean Sørensen-Dice coefficient of 0.95 for liver segmentation and 0.734 for tumor segmentation. A detailed description of image preprocessing has already been reported in the manuscript that compared different machine learning approaches using the same dataset with all 491 patients [13] and presented in supplementary material. In this analysis, in addition to prognostic value of radiomics based imaging features, treatment interactions of these features were evaluated using a single approach.

Quantitative features of tumor segmentations were computed using PyRadiomics (version 3.0.1), including shape, gray-level distribution, and texture features on the original images. In total, 100 features were derived from each segmentation. Notably, these features were extracted from a unified segmentation encompassing all liver metastases for each patient. This methodology, treating multiple intrahepatic metastases in one patient as a singular observation, especially impacting the computation and subsequent interpretation of shape-based radiomics features.

## 3 Statistical analysis

### 3.1 Model building

In this study the survival analysis with respect to the right-censored outcome was conducted using a Cox proportional hazards model. To enhance model robustness and prevent overfitting, we applied standard cross-validated LASSO (CV LASSO) regularization.

The predictors incorporated into the model were derived from both clinical data and radiomics features. Clinical data, including factors such as age, baseline disease characteristics, the presence of extra-hepatic lesions, and the sidedness of tumor origin, were considered as potential predictors of survival.

### 3.2 Treatment interactions

Furthermore, to account for potential treatment-specific effects, we introduced an interaction term for each predictor with the treatment variable, specifically the administration of radioembolization (yes/no). To evaluate whether the treatment effect on overall survival differed across levels of subgroups (categorical parameters), a Cox proportional hazards model including an interaction term between treatment and subgroup was fitted. For continuous variables, interaction with treatment was assessed by including a multiplicative interaction term between treatment and the continuous variable in the Cox model. A Wald test was used to assess the statistical significance of the interaction term. A two-sided p-value < 0.05 was considered evidence of heterogeneity of treatment effect. This approach allows the model to capture how the influence of each predictor on survival may be modified by the presence or absence of radioembolization treatment.

### 3.3 Regularization

For the selection of relevant predictors, we employed LASSO regularization, which aids in identifying the most influential predictors while mitigating the risk of overfitting. Especially, the inclusion of interaction terms with the treatment variable in the LASSO framework aimed to identify relevant interactions between predictors and treatment status, providing a comprehensive understanding of the factors influencing survival outcomes in the context of radioembolization. Confidence intervals for the estimated coefficients can not be provided due to the inherent nature of the LASSO regularization, which induces sparsity in the parameter estimates, making the traditional calculation of confidence intervals challenging and often unreliable.

### 3.4 Risk-group analysis

Furthermore, we establish risk groups by classifying instances with a predicted probability of survival less than 50% as high risk and those with a probability of 50% or greater as low risk. Subsequently, these risk groups are employed in a Kaplan-Meier plot to visually explore a potential treatment effect.

## 4 Results

Out of 530 patients randomized within SIRFLOX trial, baseline portal-venous phase CT images were available for 491 patients (333 [67.8%] male) with a median age of 63 (range, 28–83 years). 252 patients were randomized to mFOLFOX6 plus SIRT arm and 239 patients to mFOLFOX6 arm. There was no significant difference between treatment arms in terms of age, gender, WHO-PS, liver tumor ratio, in-situ primary tumor, histologic differentiation of primary tumor, previous chemotherapy, and bevacizumab therapy (Table 1). Right-sided primary tumor was more common in the mFOLFOX plus SIRT arm (27.3% vs. 20.0%, p = 0.051). There was no significant difference in overall survival of both arms (23.7 vs. 24.9 months, p = 0.68).

**Table 1:** Baseline characteristics.

	**Overall** **(n = 491)**	**mFOLFOX6** **(n = 239)**	**mFOLFOX6 +SIRT** **(n = 252)**	**p-value**
Gender (Male)	333 (67.8%)	160 (66.9%)	173 (68.6%)	0.686
Age (≥65 years)	220 (44.8%)	109 (45.6%)	111 (44.0%)	0.728
WHO-PS • 0 • 1 • Missing	326 (66.3%) 164 (33.4%) 1	158 (66.1%) 81 (33.8%) -	168 (66.6%) 83 (32.9%) 1	0.846
Site of primary tumor • Both • Left • Right • Missing	10 (2.0%) 362 (73.7%) 117 (23.8%) 2 (0.4%)	5 (2.0%) 186 (77.8%) 48 (20.0%) -	5 (1.9%) 176 (69.8%) 69 (27.3%) 2 (0.8%)	0.051*
Median liver tumor ratio, %	12.1	12.3	12	0.981
Histologic differentiation • Poor • Well • Missing	78 (15.8%) 247 (50.3%) 166 (33.8%)	41 (17.1%) 117 (48.9%) 81 (33.8%)	37 (14.6%) 130 (51.5%) 85 (33.7%)	0.423
Primary tumor, in situ	216 (43.9%)	107 (44.7%)	109 (43.2%)	0.735
Previous chemotherapy	27 (5.4%)	14 (5.8%)	13 (5.1%)	0.734
Bevacizumab	270 (54.9%)	132 (55.2%)	138 (54.7%)	0.865
Extrahepatic disease • Lung • Lung+LN • LN	73 (14.8%) 32 (6.5%) 89 (18.1%)	34 (14.2%) 17 (7.1%) 44 (18.4%)	39 (15.4%) 15 (5.9%) 45 (17.8%)	0.82
Median NLR	3.48	3.45	3.56	0.675

* Right vs. left

**Fig. 2:**
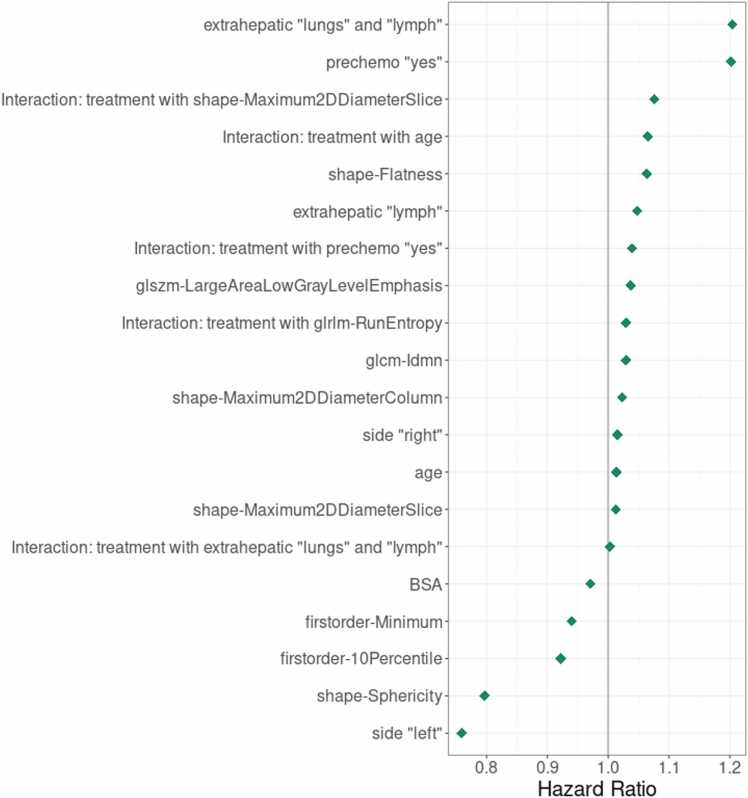
Point estimates of hazard ratios for relevant effects identified by the LASSO survival model. Confidence intervals are not shown due to the nature of the LASSO method. Hazard ratios greater than 1, indicating an increased risk associated with the respective variables.

Utilizing the Cox proportional hazards (Cox-PH) model with LASSO regularization yielded a set of n = 20 parameters correlating with overall survival. The detailed summary of all remaining and pertinent variables, along with their associated hazard ratios (HRs), is depicted in Fig. 2 and in Supplementary Table 1. Clinical parameters such as lung and lymph node metastasis, prior adjuvant chemotherapy, lymph node metastasis, right-sided tumor, and age were associated with poor survival, while BSA and left-sided tumor were associated with better overall survival. Besides those, LASSO analysis identified radiomics features including shape-Flatness, glszm-LargeAreaLowGrayLevelEmphasis, glcm-Idmn, shape-Maximum2DDiameterColumn, shape-Maximum2DDiameterSlice as poor prognostic factors, while firstorder-Minimum, firstorder-10Percentile, and shape-Sphericity as better prognostic factors.

In addition to those, LASSO analysis identified five parameters associated with additional benefit from SIRT, including radiomics parameters of shape-Maximum2DDiameterSlice and glrlm-RunEntropy, as well as clinical parameters of age, prior adjuvant chemotherapy, and lung and lymph node metastasis.

**Fig. 3:**
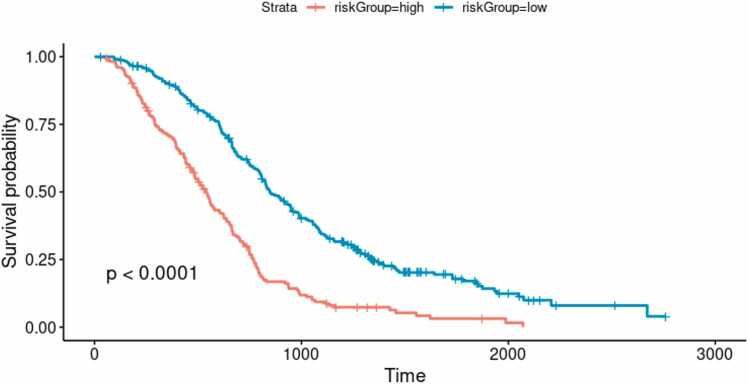
Kaplan-Meier (KM) curves for high and low risk groups based on predictions from the final model.Instances with a predicted probability of 50% or greater are classified as high risk. The p-value for the difference between the curves is calculated using the log-rank test.

Additionally, by dividing patients into high and low risk groups based on their predicted survival (> 50%: low risk; ≤ 50%: high risk), we found that the high risk group showed significantly shorter overall survival compared to the low risk group (median survival time 538 days vs. 847 days, p < 0.001; Fig. 3).

## 5 Discussion

Our study shows that radiomics-based imaging features, in addition to clinical parameters, are prognostic factors in patients with metastatic colorectal cancer treated with chemotherapy alone or combined with SIRT. The scoring system defined in this analysis based on clinical and radiomics parameters was able to identify patients with poorer outcomes. Additionally, the treatment interaction test identified some radiomics parameters as predictors of potential treatment benefit.

Systemic treatment is the primary treatment option in liver metastasis of colorectal cancer. Despite many promising initial studies [3], [14], [15], randomized trials have failed to show benefit from additional SIRT to chemotherapy [6], [7]. However, dosimetry approach has changed substantially since these trials, which employed body-surface area method with whole liver treatment approach. The whole liver approach is almost abandoned with accumulating experience in SIRT, and selective treatment is employed whenever possible, which increases the tumor dose and limits the radiation damage to healthy liver parenchyma. A multicenter study has shown that partition model-based dosimetry significantly improves overall survival compared to body surface area model [16]. Furthermore, it has been shown that tumor dose is the most important prognostic factor in colorectal cancer patients receiving SIRT, and tumor dose higher than 120 Gy increases objective response 4-fold [17]. Such improvements have led to EPOCH trial to meet its primary outcome of improved progression-free survival after additional SIRT in second-line treatment [9]. These results underline the role of SIRT in colorectal cancer, and the need for markers for better patient selection [18]. Our results showed radiomics-based imaging markers in baseline CT images are correlated with overall survival after chemotherapy alone or chemotherapy combined with SIRT in the first-line setting. Additionally, a scoring system developed based on identified clinical and imaging markers was able to differentiate patients with better outcome.

Since all colorectal cancer patients undergo imaging for staging of the disease before treatment, such imaging markers can be evaluated in each patient. Additionally, due to repeating imaging every 2–3 months to assess the treatment response and development of new lesions, radiological images offer the possibility to re-evaluate the radiomics-based prognostic and predictive factors at each follow-up. Considering the invasiveness, technical difficulty, and risk of sampling error of tumor biopsy, imaging parameters offer a more suitable alternative for the evaluation of spatial and temporal tumor heterogeneity. Additionally, histopathological evaluation is not usually pursued in patients with metachronous liver metastasis; however, it has been shown that clinical parameters crucial for treatment decision making shows heterogeneity between primary tumor and metastatic lesions, such as KRAS mutation status [19]. Previous studies showed that the radiomics analysis of liver metastases in CT or MRI can identify RAF and BRAF mutation status in patients with colorectal cancer [20], [21]. Also, some studies have shown the prognostic value of radiomics-based markers in metastatic colorectal cancer patients treated with systemic agents [22], [23], [24], [25]. Additionally, a recent preliminary analysis showed the prognostic role of radiomics in 51 patients with colorectal cancer treated with radioembolisation [26]. These results underline the potential role of radiomics-based imaging parameters in the clinical decision making of colorectal cancer treatment.

In addition to prognostic value of these markers, our study identified the predictive value of radiomics-based markers of shape-Maximum2DDiameterSlice and glrlm-RunEntropy. Biologically, the shape-Maximum2DDiameterSlice metric likely reflects lesion size and geometry; larger tumors tend to harbour more central necrosis, variable perfusion, and hypoxic regions, which can alter microsphere deposition and radiosensitivity. glrlm-RunEntropy, which captures intratumoral textural randomness, is associated with heterogeneity in density/contrast, which has been linked to variable cellularity, necrosis and changes in perfusion. Such heterogeneity has repeatedly been associated with differential responses to chemotherapy and SIRT in liver metastases. Together, these imaging features may act as non-invasive surrogates for microenvironmental (perfusional/hypoxic) and biological (cellularity/genomic heterogeneity) properties that modulate the efficacy of both chemotherapy and SIRT. A prognostic factor is a marker that correlates with the course of the disease independent of the therapy, but a predictive factor shows a specific subgroup with a high likelihood of benefiting from an individual treatment [27]. Therefore, predictive factors can be evaluated only in studies with a control arm. Although SIRFLOX was a negative study with no difference in overall survival between treatment arms (23.7 vs. 24.9 months), these markers were able to predict potential benefit from additional SIRT. Although it was not planned in this study, considering the increasing knowledge in that area [28], delta-radiomics analyses in clinical trial datasets can improve the patient stratification using imaging datasets.

This study has some limitations. It was a post-hoc analysis of a randomized controlled trial conducted in 87 centers, which leads to variations in scanners, imaging protocols, and timing of portal-venous phase images. Furthermore, automated segmentation model training was done only by manual segmentations of a single radiologist. However, this offers the possibility to reach a sample size of 491 patients and shows that radiomics-based markers can be used in a relatively heterogeneous dataset. Also, to overcome the intra-patient tumor heterogeneity, all liver lesions were segmented and evaluated. Using all segmented liver metastases enabled a comprehensive characterization of intrapatient tumor heterogeneity and total tumor burden, which may provide a more accurate reflection of systemic disease biology. However, this approach introduces potential variability in some features, particularly related to lesion shape, and requires appropriate aggregation of features at the patient level to avoid pseudoreplication. Conversely, restricting the analysis to a single (i.e. the largest) lesion enhances feature robustness and statistical simplicity, but could lead to an underestimation of biological heterogeneity. Fitting prognostic or predictive models to high-dimensional data with a very large number of potential predictors bears the risk of overfitting the models. In the present study, this risk was mitigated by using the regularized and well-established LASSO approach. Additionally, the developed scoring system has not been externally validated. However, considering the need for control arm to evaluate predictive value of radiomics-based markers, it was not possible to validate the system externally.

## 6 Conclusions

Radiomics-based imaging markers using pretreatment CT images are prognostic factors in colorectal cancer patients with liver metastasis treated with chemotherapy alone or combined with SIRT in the first-line setting. Scoring system based on clinical and radiomics-based markers can be used to identify patients with poor outcome. These markers are also predictive factors correlating therapy outcome.

## Abbreviations

Cox-PH, Cox Proportional Hazards; CT, Computed Tomography; CV LASSO, Cross-Validated Least; Absolute Shrinkage and Selection Operator; FOLFOX, Folinic Acid (Leucovorin), Fluorouracil (5-FU), and Oxaliplatin; FOLFIRI, Folinic Acid (Leucovorin), Fluorouracil (5-FU), and Irinotecan; hPFS, Hepatic Progression-Free Survival; HR, Hazard Ratio; LASSO, Least Absolute Shrinkage and Selection Operator; PFS, Progression-Free Survival; RECIST 1.1, Response Evaluation Criteria in Solid Tumors, version 1.1; SIRT, Selective Internal Radiation Therapy; WHO-PS, World Health Organization Performance Status

## CRediT authorship contribution statement

**Max Seidensticker:** Writing – review & editing, Writing – original draft, Validation, Supervision, Project administration, Methodology, Formal analysis, Data curation, Conceptualization. **Osman Öcal:** Writing – review & editing, Writing – original draft, Visualization, Project administration, Methodology, Investigation, Formal analysis, Data curation, Conceptualization. **Moritz Wildgruber:** Writing – review & editing, Project administration, Methodology, Formal analysis, Conceptualization. **Nabeel Mansour:** Writing – review & editing, Methodology, Formal analysis, Data curation. **Anna Theresa Stüber:** Writing – review & editing, Software, Methodology, Investigation, Formal analysis, Data curation. **Abaci Gizem:** Writing – review & editing, Methodology, Investigation, Data curation. **Matthias Philipp Fabritius:** Writing – review & editing, Investigation, Formal analysis, Data curation. **Jens Ricke:** Writing – review & editing, Writing – original draft, Supervision, Project administration, Data curation, Conceptualization. **Sinan Deniz:** Writing – review & editing, Software, Investigation, Formal analysis, Data curation. **Daniel Puhr-Westerheide:** Writing – review & editing, Software, Formal analysis, Data curation. **Michael Ingrisch:** Writing – review & editing, Supervision, Methodology, Formal analysis, Data curation, Conceptualization.

## Ethical statement

The local ethics committees approved the study. Study procedures were performed per the study protocol. All patients provided written, informed consent to participate in the study (SIRFLOX trial). All applicable institutional and/or national guidelines for the care and use of animals were followed.

## Funding

This research did not receive any specific grant from funding agencies in the public, commercial, or not-for-profit sectors.

## Declaration of Competing Interest

The authors do not have any personal financial interests related to the subject matters discussed in this manuscript.
