# Supplementary material for: Prognostic and predictive value of radiomics-based imaging features in patients with colorectal liver metastasis receiving radioembolisation in first-line setting
Source: Eur J Radiol Open. 2026 Apr 15;16:100750. doi: 10.1016/j.ejro.2026.100750 (PMC13096897; doi:10.1016/j.ejro.2026.100750)
Supplement: Supplementary file 1 — Supplementary material [file mmc1.docx]

**CT Image Acquisition Guidelines (***modified from J Clin Oncol 34, 1723-1731(2016)***)**

CT images are to be acquired from the lung apices to the symphysis pubis.

Patient Orientation: Supine

Scan Location / Coverage: Lung apices through symphysis pubis

Breathing Instructions: Single breath-hold

Time per Tube rotation: 1 second or less

Scan FOV: Large, enclosing the cross section of the whole body (consistent throughout the study)

Display FOV: Unique to patient size

CT specs (no. of slices): Multidetector CT strongly preferred (single slice CT, only if unavoidable)

Reconstruction Kernel: Image reconstruction should be made with an appropriate kernel for lung and soft tissue (mediastinum, abdomen, pelvis) in axial orientation

Only CT images post contrast are required for the SIRFLOX study. Please provide CT

images in the following post contrast phases:

- portal-venous: liver (ensure high quality)
- venous: abdomen, pelvis
- any time post-contrast: lung

Details of contrast media application:

IV Contrast Required, 80-160 mL, non-ionic only

IV Contrast Concentration 150-370 mg/ml (300-370 mg/ml preferred)

Injection Rate Single injection: 2-5 cc/sec (power injector)

Oral Contrast Preferred, use standard protocol

Sequence parameters:

Please use parameters as close as possible to those listed below. Once the preferred

parameters have been selected, they should remain consistent throughout the baseline

screening and response assessment stages of the study.

Sequence parameters: Liver

IV Contrast Portal-venous phase (ensure high quality)

Slice Thickness 2 mm preferred (up to 5 mm accepted)

Reconstruction Interval Smaller than or equal to slice thickness

Gap None (i.e., contiguous or overlapping reconstruction)

Orientation Axial required

Sequence parameters: Abdomen / pelvis

IV Contrast Venous / equilibrium required

Slice Thickness 2 mm preferred (up to 5 mm accepted)

Reconstruction Interval Smaller than or equal to slice thickness

Gap None (i.e., contiguous or overlapping reconstruction)

Orientation Axial required

**Image preprocessing**

The dataset primarily consists of axial-oriented image slices, but some coronal CTs are blended in, requiring filtering. After cleansing, the dataset contains 491 samples, which are converted to NIfTI format for convenience. This format stores all CT slices in a single file, bundles meta-information, and converts the CT image data to a valid Hounsfield unit scale automatically. Liver and tumor segmentations are obtained using a trained nn-Unet to extract relevant information from abdominal CTs. Around 80 expert segmentations are available to evaluate the quality of this automatic process, with a mean Sørensen-Dice coefficient of 0.95 for liver segmentation and 0.734 for tumor segmentation.

The preprocessing involves resampling, such that each voxel represents 1 mm³ across the dataset. The liver segmentation is not only used as a mask but also to locate the liver in the abdominal scan, and the complete scan is cropped to a 240 mm³ cube that contains the liver completely. This cube is then scaled down to 64 × 64 × 64 to match the expected input size for the network architecture. The downscaling from an original resolution of 512 is intentionally desired due to the low number of samples and the immense computational effort in 3D-CNNs. However, this may result in the loss of important aspects. The pixel intensities on the Hounsfield scale are clamped to (10, 150), as the area of interest contains only soft tissue. Subsequently, min-max normalization is applied to map the image arrays into pixel space. The final training input is a two-channel volumetric tensor, with the first channel corresponding to the actual masked CT data and the second channel representing an overlay of the segmentation.
