# Supplementary material for: Prognostic and predictive value of radiomics-based imaging features in patients with colorectal liver metastasis receiving radioembolisation in first-line setting
Source: Eur J Radiol Open. 2026 Apr 15;16:100750. doi: 10.1016/j.ejro.2026.100750 (PMC13096897; doi:10.1016/j.ejro.2026.100750)
Supplement: Supplementary file 2 — Supplementary material [file mmc2.docx]

**Supplementary table 1. HRs for n = 20 features selected with LASSO model**

|  | **Feature** | **beta / effect** | **exp(beta)** |
| --- | --- | --- | --- |
| 1 | *age* | 0.013324 | 1.0134 |
| 2 | *BSA* | -0.029502 | 0.9709 |
| 3 | *side "left"* | -0.275797 | 0.7590 |
| 4 | *side "right"* | 0.015078 | 1.0152 |
| 5 | *prechemo "yes"* | 0.183846 | 1.2018 |
| 6 | *extrahepatic "lymph"* | 0.046766 | 1.0479 |
| 7 | *extrahepatic "lungs" and "lymph"* | 0.185704 | 1.2041 |
| 8 | *shape-Flatness* | 0.061617 | 1.0636 |
| 9 | *shape-Maximum2DDiameterColumn* | 0.022840 | 1.0231 |
| 10 | *shape-Maximum2DDiameterSlice* | 0.012376 | 1.0125 |
| 11 | *shape-Sphericity* | -0.226832 | 0.7971 |
| 12 | *firstorder-10Percentile* | -0.081237 | 0.9220 |
| 13 | *firstorder-Minimum* | -0.062072 | 0.9398 |
| 14 | *glcm-Idmn* | 0.028607 | 1.0290 |
| 15 | *glszm-LargeAreaLowGrayLevelEmphasis* | 0.036184 | 1.0368 |
| 16 | *Interaction: treatment with age* | 0.062991 | 1.0650 |
| 17 | *Interaction: treatment with prechemo "yes"* | 0.037951 | 1.0387 |
| 18 | *Interaction: treatment with extrahepatic "lungs" and "lymph"* | 0.002603 | 1.0026 |
| 19 | *Interaction: treatment with shape-Maximum2DDiameterSlice* | 0.073252 | 1.0760 |
| 20 | *Interaction: treatment with glrlm-RunEntropy* | 0.028703 | 1.0291 |
